# Supplementary material for: Resolving the full spectrum of human genome variation using Linked-Reads
Source: Genome Res. 2019 Apr;29(4):635–45. doi: 10.1101/gr.234443.118 (PMC6442396; doi:10.1101/gr.234443.118)
Supplement: Supplemental Material [file supp_gr.234443.118_Supplemental_longranger-2.2.zip › longranger-2.2.tar.bz2!/lr2.2-public/lib/stan/cmdstan/stan/doc/api/html/statement__2__grammar_8hpp_source.html]

Stan: src/stan/gm/grammars/statement\_2\_grammar.hpp Source File


|  |  |
| --- | --- |
|  | Stan  2.5.0  probability, sampling & optimization |


- Main Page
- Related Pages
- Namespaces
- Classes
- Files

- File List
- File Members

All Classes Namespaces Files Functions Variables Typedefs Enumerations Enumerator Friends Macros Pages

- src
- stan
- gm
- grammars

statement\_2\_grammar.hpp

Go to the documentation of this file.

1 #ifndef STAN\_\_GM\_\_PARSER\_\_STATEMENT\_2\_GRAMMAR\_\_HPP

2 #define STAN\_\_GM\_\_PARSER\_\_STATEMENT\_2\_GRAMMAR\_\_HPP

3

4 #include <string>

5 #include <sstream>

6 #include <vector>

7

8 #include <boost/spirit/include/qi.hpp>

9

10 #include <stan/gm/ast.hpp>

11 #include <stan/gm/grammars/whitespace\_grammar.hpp>

12 #include <stan/gm/grammars/expression\_grammar.hpp>

13

14 namespace stan {

15

16  namespace gm {

17

18  template <typename Iterator>

19  struct statement\_grammar;

20

21  template <typename Iterator>

22  struct statement\_2\_grammar

23  : boost::spirit::qi::grammar<Iterator,

24  statement(bool,var\_origin,bool),

25  whitespace\_grammar<Iterator> > {

26

27

28

29  statement\_2\_grammar(variable\_map& var\_map,

30  std::stringstream& error\_msgs,

31  statement\_grammar<Iterator>& sg);

32

33

34  // global info for parses

35  variable\_map& var\_map\_;

36  std::stringstream& error\_msgs\_;

37

38  // grammars

39  expression\_grammar<Iterator> expression\_g;

40  statement\_grammar<Iterator>& statement\_g;

41

42  // rules

43

44  boost::spirit::qi::rule<Iterator,

45  conditional\_statement(bool,var\_origin,bool),

46  whitespace\_grammar<Iterator> >

47  conditional\_statement\_r;

48

49

50  boost::spirit::qi::rule<Iterator,

51  statement(bool,var\_origin,bool),

52  whitespace\_grammar<Iterator> >

53  statement\_2\_r;

54

55  };

56

57

58  }

59 }

60

61 #endif

stan::gm::statement\_2\_grammar::var\_map\_

variable\_map & var\_map\_

**Definition:** statement\_2\_grammar.hpp:35

stan::gm::statement

**Definition:** ast.hpp:634

stan::gm::statement\_2\_grammar::statement\_2\_grammar

statement\_2\_grammar(variable\_map &var\_map, std::stringstream &error\_msgs, statement\_grammar< Iterator > &sg)

**Definition:** statement\_2\_grammar\_def.hpp:88

stan::gm::statement\_2\_grammar::statement\_2\_r

boost::spirit::qi::rule< Iterator, statement(bool, var\_origin, bool), whitespace\_grammar< Iterator > > statement\_2\_r

**Definition:** statement\_2\_grammar.hpp:53

stan::gm::statement\_2\_grammar::error\_msgs\_

std::stringstream & error\_msgs\_

**Definition:** statement\_2\_grammar.hpp:36

stan::gm::statement\_grammar

**Definition:** statement\_2\_grammar.hpp:19

stan::gm::statement\_2\_grammar

**Definition:** statement\_2\_grammar.hpp:22

stan::gm::conditional\_statement

**Definition:** ast.hpp:729

expression\_grammar.hpp

stan::gm::expression\_grammar

**Definition:** expression07\_grammar.hpp:23

stan::gm::statement\_2\_grammar::conditional\_statement\_r

boost::spirit::qi::rule< Iterator, conditional\_statement(bool, var\_origin, bool), whitespace\_grammar< Iterator > > conditional\_statement\_r

**Definition:** statement\_2\_grammar.hpp:47

stan::gm::variable\_map

**Definition:** ast.hpp:439

stan::gm::statement\_2\_grammar::expression\_g

expression\_grammar< Iterator > expression\_g

**Definition:** statement\_2\_grammar.hpp:39

stan::gm::statement\_2\_grammar::statement\_g

statement\_grammar< Iterator > & statement\_g

**Definition:** statement\_2\_grammar.hpp:40

ast.hpp

stan::gm::whitespace\_grammar

**Definition:** whitespace\_grammar.hpp:11

whitespace\_grammar.hpp

stan::gm::var\_origin

int var\_origin

**Definition:** ast.hpp:411


---

[ Stan Home Page ]

*© 2011–2014,
Stan Development Team.*
